# Co-exposures to physical and psychosocial work factors increase the occurrence of workplace injuries among French care workers

**DOI:** 10.3389/fpubh.2022.1055846

**Published:** 2022-12-13

**Authors:** Régis Colin, Pascal Wild, Christophe Paris, Stéphanie Boini

**Affiliations:** ^1^Department of Occupational Epidemiology, Occupational Health and Safety Institute (INRS), Vandoeuvre-les-Nancy, France; ^2^INSERM U1085 Institut de Recherche en Santé, Environnement et Travail (IRSET), Rennes, France

**Keywords:** workplace injuries, physical factors, psychosocial factors, co-exposure, care workers

## Abstract

**Objective:**

The aim of this study was to analyze the effect of co-exposures to physical and psychosocial factors (PSF) regarding the incidence of workplace injuries (WI) among care workers. Additional objective was to identify the work factors associated with the co-exposure combinations leading to the highest rates of WI.

**Methods:**

The study sample consisted of 4,418 care workers participating to the French Working Conditions Survey both in 2013 and 2016. WI were assessed during the 4-year follow-up by matching the databases of the National Health Insurance Funds' compensation system. We assessed exposure for physical factors and PSF using factorial analyses and hierarchical clustering. We implemented a Poisson regression model with the WI incidence as the outcome and the clusters as independent variables of interest. Logistic regression model allowed identifying the work factors that predicted co-exposure combinations with a WI rate > 40%.

**Results:**

WI were highly related to both physical and psychosocial exposures. With low exposure to one or the other, there was no increased risk of WI. Physical factors and PSF potentiated each other and their co-exposure significantly increased the risk of WI, with model predicted rates per 1,000 persons-year for those most exposed to physical risk of 14.6 [4.5–24.8] with low PSF and 38.0 [29.8–46.3] with high PSF. Work factors that predicted co-exposure combinations with a rate > 40 WI% were: working as nursing assistant or hospital services officer, lack of predictability and flexibility of schedules, overtime, controlled schedules, work-family imbalance and insufficient preventive measures.

**Conclusions:**

Our findings highlight the need to take into account psychosocial factors in addition of only considering physical factors when analyzing WI occurrence, as usually done. Prevention actions must be taken to reduce both physical and psychosocial exposure. These results provide keys points for the prevention of WI among care workers.

## Introduction

Worldwide, the population of care workers is increasing, and they are victims of many workplace injuries (WI). For example, in United States, the rate of injury for emergency medical technicians and paramedics was about three times the national average for all occupations (1). Among care workers, the highest WI rates were observed among nursing professions (2). An Australian retrospective cohort analysis observed that health and social care workers had a significantly higher rate of WI than workers in other industries and found the highest rate of WI for ambulance officers and social workers (3). Similarly in France, the official statistics reported 3.5 million care workers (10% more than 10 years ago) in 2019. This year, 180,000 WI were recorded for this population. With a WI rate of 52.3 per 1,000 care workers, they rank first among injured workers, ahead of the construction industry and well above the national average (33.5 WI per 1,000 workers) (4). The rate of WI among care workers has increased by 12% in 5 years, while that of the other types of work activities has followed a downward trend.

The notion of “care work” covers the activities of care given to others, from the youngest to the oldest (medical care, dependence, meals, living space, wellbeing, etc.) and constitutes a concrete response to the needs of others. This occupational sector includes professions as varied as home care aide, nurse, laboratory technician, dental assistant, surgeon, etc., carried out in different contexts such as hospitals, the home, housing for elderly dependent persons, nursing homes, town practices, etc. They are subject to multiple occupational risks. Their function makes them particularly prone to musculoskeletal disorders (5–7) and low back pain (5, 8). All care workers have in common that they are exposed to a wide range of occupational risks related to physical activity mostly attributable to manual handling (9), falls and slips (10), and infectious (11), biological (12) and chemical risks [disinfectants (13), anesthetic gases (14), drugs (15)]. Because of the necessity of continuity of care, they are subject to a specific work organization that regularly requires shift work, night work (12, 16) and long and compressed work schedules (17). They are also particularly exposed to psychosocial risk factors (PSF) [stressful work organization (2), confrontation with illness, end of life, death (18), violence from colleagues (19, 20) or from patients and their relatives (21)].

Longitudinal studies that have observed the effect of PSF on the occurrence of WI for care workers are however scarce (22). In 2019, a prospective cohort study investigated the risk factors for back injury in the healthcare sector in Denmark and concluded that in addition to physical burden, poor collaboration between and support from colleagues increased the risk of back injury (23). A Dutch study found that low autonomy and exposure to harassment and violence inside and outside the organization were associated with WI for workers in the health and welfare sector (20).

Regarding the co-exposure between physical and psychosocial factors, a cross-sectional study found a higher prevalence of patient-handling injury when nursing personnel declared both high physical demand and low decision latitude (24). A recent longitudinal study found that the difference in WI rates between high and low psychosocial exposures seemed to increase with increasing physical exposure among workers from all sectors of activity, but not only care workers (25). To our knowledge, no prospective study has yet focused on the effects of joint exposure to physical factors and PSF on the occurrence of WI among care workers.

The literature, as described above, highlights the lack of prospective studies analyzing the effect of psychosocial factors on the occurrence of WI in care workers. Workers exposed to a single occupational risk factor are rare and are in fact most often exposed to several risk factors simultaneously. Multi-exposures between the risk factors for WI, and in particular between physical and psychosocial risk factors, have been scarcely explored. However, considering the synergetic effects between exposure to physical and psychosocial risks in the occurrence of WI seems to be a plausible hypothesis in order to identify the determinants for better-adapted prevention.

The objective of this study was, first, to analyse the effect of co-exposures to physical and psychosocial factors regarding the incidence of workplace injury among care workers. Secondly, our study aimed to identify the work factors associated with the co-exposure combinations leading to the highest rates of WI.

## Methods

### Study design

This observational study was designed as a prospective cohort study nested in the French Working Conditions Survey, with a follow-up period of 4 years. Participants of this study were workers interviewed in both 2013 and 2016.

### Setting and participants

In the French Working Conditions Survey, trained interviewers questioned 33,673 workers face-to-face in 2013. In 2016, 22,852 of them were again interviewed. Data were collected from workers on working hours and the organization of working time, organization and the pace of work, risks, hardships and their prevention, psychosocial constraints, relations with the public and violence at work. Using the participants' social security ID (if available), the survey data were matched with data relative to WI, occupational diseases and the consumption of medical care or treatments, obtained from the databases of the National Health Insurance compensation system.

Study population consists of 17,831 workers interviewed in both 2013 and 2016. The exclusion criteria were: not providing a social security ID (*n* = 2,665); not being registered in the National Health Insurance compensation system (*n* = 2,337); and not having sufficient information about employment (*n* = 19).

The present paper focuses on care workers. We considered as a care worker the respondents reporting that their main function in their job was to care for people or that their main profession featured on a list of selected professional activities (nursing assistant, nurse, midwife, medical and paramedical profession, social workers, professional of cultural or sports activities, home care, nursery assistant and hospital services officer). Finally, our study sample consisted of 4,418 care workers.

### Outcome: Workplace injuries

The claims for recognition of WI are recorded by the National Health Insurance. WI were assessed during the 4-year follow-up. Detailed characteristics were collected on the date of occurrence, the nature and the site of the injury, and the duration of sick leave. The main outcome was the occurrence of a WI during the follow-up period, whatever the nature or duration of the sick leave as recorded by the National Health Insurance.

### Risk factors

Numerous risk factors were investigated in this study. Given the large number of questions, we grouped them into broad categories or subgroups of physical factors and psychosocial factors identified by a group of French experts (26). The process to characterize physical and PSF exposures is detailed in Supplementary material 1.

#### Physical factors

We grouped the 20 items related to physical risk factors into six categories (Supplementary material 1):

- Awkward or uncomfortable postures (standing for long periods of time, staying in another awkward or tiring posture for a long time, performing painful or tiring movements, making long or frequent trips on foot);
- Carrying heavy loads (does your job require you to carry or move heavy loads?);
- Vibration or shaking (does your job cause you to shake or vibrate?);
- Loud noise (can you hear a person placed 2 or 3 meters away from you when they speak to you?);
- Concentration (keeping your eyes on the work, reading small, poorly printed, poorly written letters or numbers, examining very small objects, small details, reading and paying attention to brief, unpredictable, or hard-to-detect visual or audible signals);
- Unhealthy work environment (dirt, humidity, drafts, bad smells, high temperature, low temperature, lack or poor condition of sanitary facilities, no view of the external environment, lack of privacy).

#### Psychosocial factors

The following six groups were defined based on the 98 items (Supplementary material 1):

- Labor intensity and working time (three subgroups: excessive workload, time pressure, work complexity);
- Emotional demand (four subgroups: contact with suffering, poor relationship with the public, emotional dissonance, fear for safety during work);
- Lack of autonomy (five subgroups: monotony and boredom, lack of pleasure at work, skills not fully utilized or developed, unpredictability, no choice of how a job is done);
- Social relationships at work (10 subgroups: violence, poor cooperation between colleagues and integration in a team, poor team autonomy and work engagement, lack of support from superiors, lack of leadership, lack of organizational justice, lack of reward, lack of career prospects, insufficient salary, inadequate social recognition of the job);
- Conflict of values (two subgroups: ethical conflicts, no opportunity to perform high-quality jobs);
- Job insecurity (three subgroups: job instability, lack of work sustainability, occupational changes).

### Other covariates

The questionnaire also collected items on socio-demographic characteristics: gender, age, educational level, family structure and monthly income per consumption unit. Furthermore, information about Health included sleep problems and the use of psychotropic drugs. Finally, occupational factors included the number of workers in the company, the type of work contract, seniority in the job, the type of workplace, and occupational category.

### Statistical analysis

We calculated the mean rates of WI per 1,000 person-years (py) at work, taking into account part-time work.

We analyzed the occurrence of WI during the follow-up period as a function of the risk factors using multiple Poisson regression models accounting for the person-years at work. The models were fitted with covariates selected based on both scientific evidence and statistical considerations (stepwise selection). The results were presented in the form of a model predicted rate of WI per 1,000 py, with 95% confidence intervals and the *p*-value of the incidence rate ratio.

#### WI and exposures to each physical and each psychosocial factors

We fitted one model for each of the 6 physical exposures as well as for each of the 6 PSF exposures, as defined in Supplementary material 1; first step. The models were adjusted for gender, age class, educational level, work contract, seniority, sleep problem, and use of psychotropic drugs. In addition, we considered models with interaction between the risk factors and gender.

#### WI and co-exposures to physical and psychosocial factors

We used hierarchical clustering on the six categories of physical factors and the six categories of PSF, resulting in 3 levels of physical exposure and three levels of PSF exposure, respectively (Supplementary material 1, second step). We fitted the profiles of physical exposure and PSF exposure in interaction, adjusting for the previous covariates.

#### Work factors associated with the co-exposure combinations leading to the highest WI rates

Within each pairwise combination of the six physical categories and the six psychosocial categories, we computed model-based rates of WI according to the detailed exposure levels. We thus identified the pairwise combinations of physical and psychosocial factors with a rate higher than 40 WI per 1,000 py (subsequently called “high-rate group”). We modeled the probability of this high-rate group according to work activity, and sociodemographic and organization characteristics using logistic regression with a backward-stepwise selection.

In order to check the fit of the model, we first calculated the model-based area under the ROC curve. Secondly, we checked the relevance of the predicted probability to belong to this high-rate group by performing a logistic regression with the occurrence of WI as the outcome.

We performed the statistical analysis using Stata version 15.1 (StataCorp. LLC, Tx, USA), except for the clustering process, which was performed using R (27). The level of statistical significance was set at 0.05.

## Results

### Characteristics of the study population

The population of interest included 4,418 care workers (25% of the study population), mostly women (84%) with a mean age of 43 years in 2013 (Table 1). Sixty-three percent of them had an educational level higher than the secondary school certificate. Their family composition and income were not different from other workers. With regard to health, 34% reported having sleep problems and 11% were using psychotropic drugs. A majority of care workers worked in a company with more than 50 employees, and had more than 5 years' seniority at the time of inclusion (Table 1).

**Table 1:** Characteristics of participants at inclusion in 2013 and mean rate of workplace injuries (WI).

	**Care workers**				**Other workers**				**All workers**			
	**(*N* = 4,418)**			**Mean**	**(*N* = 13,413)**			**Mean**	**(*N* = 17,831)**			**Mean**
	***n***	**%**	**WI**	**rate of WI^a^**	***n***	**%**	**WI**	**rate of WI^a^**	***n***	**%**	**WI**	**rate of WI^a^**
**Sociodemographic**												
**Gender**												
Male	699	15.8	43	15.9	7,107	53.0	625	22.5	7,806	43.8	668	21.9
Female	3,719	84.2	301	22.7	6,306	47.0	300	13.1	10,025	56.2	601	16.6
**Age (years)**												
≤ 30	626	14.2	66	29.0	1,776	13.2	172	25.7	2 402	13.5	238	26.5
30–50	2,364	53.5	173	19.8	7,338	54.7	520	18.2	9,702	54.4	693	18.5
≥50	1,428	32.3	105	21.4	4,299	32.1	233	15.1	5,727	32.1	338	16.6
**Educational level**												
No diploma/lower education	548	12.4	58	31.9	2,185	16.3	220	28.5	2,733	15.3	278	29.2
CAP/BEP certificates	1,107	25.1	108	27.7	3,653	27.2	403	29.4	4,760	26.7	511	29.0
Baccalaureate	632	14.3	61	26.5	2,524	18.8	175	18.2	3,156	17.7	236	19.8
Higher education	2,131	48.2	117	14.8	5,051	37.7	127	6.4	7,182	40.3	244	8.8
**Family structure**												
Alone/one-parent family/with ascendant	1,012	22.9	102	27.8	2,850	21.2	193	18.0	3,862	21.7	295	20.5
With a partner/nuclear family	3,406	77.1	242	19.7	10,563	78.8	732	18.3	13,969	78.3	974	18.6
**Monthly income per consumption unit (**€**)**												
< 1,200	798	18.1	89	34.6	2,533	18.9	266	29.7	3,331	18.7	355	30.8
1,200–1,700	1,417	32.1	130	25.2	4,172	31.1	363	22.7	5,589	31.3	493	23.4
1,700–2,200	982	22.2	61	16.5	2,814	21.0	167	15.3	3,796	21.3	228	15.6
≥2,200	1,088	24.6	52	12.9	3,467	25.8	98	7.3	4,555	25.6	150	8.6
Missing	133	3.0	12		427	3.2	31		560	3.1	43	
**Health**												
**Sleep problem** ^b^												
No	2,912	65.9	194	18.3	9,413	70.2	610	17.0	12,325	69.1	804	17.3
Yes	1,506	34.1	150	28.0	4,000	29.8	315	21.2	5,506	30.9	465	23.0
**Use of psychotropic drugs**												
No	3,956	89.5	289	20.1	12,422	92.6	838	17.7	16,378	91.9	1,127	18.3
Yes	462	10.5	55	34.6	991	7.4	87	24.5	1,453	8.1	142	27.6
**Work activity**												
**Number of workers in the company**												
< 10	797	18.0	35	12.7	2,736	20.4	174	17.9	3,533	19.8	209	16.7
10–49	726	16.4	62	24.7	3,370	25.1	303	23.7	4,096	23.0	365	23.9
50–499	1,361	30.8	189	38.2	4,646	34.6	330	18.3	6,007	33.7	519	22.6
≥500	1,323	30.0	45	9.0	2,178	16.2	101	11.8	3,501	19.6	146	10.8
Missing	211	4.8	13		483	3.6	17		694	3.9	30	
**Work contract**												
Open-ended contract	3,934	89.0	306	21.3	12,055	89.9	806	17.4	15,989	89.7	1,112	18.3
Fixed-term or temporary contract	484	11.0	38	24.6	1 358	10.1	119	26.6	1,842	10.3	157	26.1
**Seniority (years)**												
≤ 1	251	5.7	30	38.4	1 123	8.4	108	27.9	1,374	7.7	138	29.7
1–5	872	19.7	89	28.7	2,419	18.0	232	25.9	3,291	18.5	321	26.6
5–10	872	19.7	73	23.0	2,402	17.9	198	21.3	3,274	18.3	271	21.7
≥10	2,423	54.8	152	17.1	7,469	55.7	387	13.5	9,892	55.5	539	14.4
**Workplace**												
At home	288	6.5	–	–	88	0.7	–	–	376	2.1	11	8.1
Usual establishment	3,418	77.4	272	21.6	10,298	76.8	627	16.0	13,716	76.9	899	17.3
Business travel, construction site, private homes	521	11.8	60	38.1	2,422	18.1	280	31.0	2,943	16.5	340	32.1
Others	191	4.3	–	–	605	4.5	19	6.9	796	4.5	19	6.6
**Occupational category**												
Farmers, farm workers, growers	–	–	–	–	–	–	–	–	44	0.3	–	–
Craftsmen, merchants, business manager	–	–	–	–	–	–	–	–	306	1.7	10	9.1
Executives and higher intellectual professions	357	8.1	–	–	2,604	19.4	–	–	2,961	16.6	43	3.7
Intermediate professions	1,931	43.7	127	17.8	3,473	25.9	162	12.0	5,404	30.3	289	14.0
Employees	2,114	47.9	213	28.6	3,674	27.4	197	14.8	5,788	32.5	410	19.8
Blue collar workers	13	0.3	0	0.0	3,307	24.6	515	41.4	3,320	18.6	515	41.3
Missing	–	–	–	–	–	–	–	–	8	0.1	–	–

a Mean rate of workplace injuries per 1,000 py: (number of workplace injuries/person-years*1,000) weighted by part-time work.

b Difficulties to fall asleep, night waking, early awakening without being able to fall asleep again.

– Too small to mention.

### Workplace injuries

The National Health Insurance compensation system identified 344 care workers declaring at least one WI (8%) (Table 2). Sick leave for the occurrence of the first WI resulted in more than 1 day off work for 68% of them. The most frequent sites of WI were upper limb (30%), back (29%) and to a lesser extent lower limb (20%). Care workers were mainly victims of back pain, lumbago, neck pain, spine, sciatica, concussion and internal trauma (Table 2). The mean rates of WI were highest among women, the youngest, the least qualified, those with the lowest incomes, the least seniority, and precarious work contracts (Table 1—last column).

**Table 2:** Description of workplace injuries (WI) occurred in the follow up period for the entire study population and for care workers.

	**Care workers**		**Other workers**		**All workers**	
	***n***	**%**	***n***	**%**	***n***	**%**
	4,418	24.8	13,413	75.2	17,831	100.0
Males	699	15.8	7,107	53.0	7,806	43.8
Females	3,719	84.2	6,306	47.0	10,025	56.2
At least one WI during the period	344	7.8	925	6.9	1,269	7.1
At least one WI with more than 1 day off work	232	67.4	689	74.5	921	72.6
**Duration of sick leave in days** ^**a**^						
0 day	112	32.5	236	25.5	348	27.4
1–7 days	58	16.9	200	21.6	258	20.3
8 days to 1 month	106	30.8	305	33.0	411	32.4
More than 1 month	68	19.8	184	19.9	252	19.9
**Lesion site** ^**a**^						
Upper limb	103	29.9	319	34.5	422	33.3
Back	101	29.4	165	17.8	266	21.0
Lower limb	67	19.5	219	23.7	286	22.5
Head	23	6.7	91	9.8	114	8.9
Neck	17	4.9	26	2.8	43	3.4
Chest and organs	16	4.6	40	4.3	56	4.4
Whole body, multiple locations and unknown	17	5.0	65	7.0	82	6.5
**Type of injury** ^**a**^						
Concussion, internal trauma	70	20.4	233	25.2	303	23.9
Back pain, lumbago, neck pain, spine, sciatica	92	26.8	163	17.6	255	20.1
Wounds	38	11.1	216	23.4	254	20.0
Dislocation, sprain, strain	41	11.9	116	12.5	157	12.4
Traumatic shocks	31	9.0	67	7.2	98	7.7
Fracture	16	4.6	43	4.6	59	4.7
Burns, frostbite	7	2.0	19	2.1	26	2.0
Psychological trauma, violence	8	2.3	16	1.7	24	1.9
Poisoning, infection, blood exposure accident	19	5.5	3	0.3	22	1.7
Multiple wounds, other or unknown	22	6.4	49	5.3	71	5.6

a Concerns the first WI occurred in the period in case of several WIs.

The model predicted rates of WI per 1,000 py for each adjustment variable selected after a stepwise selection (sociodemographic, health, activity) are presented in Supplementary material 2.

### Physical risk factors and workplace injuries

Four out of six physical factors were significantly associated with the incidence of WI: awkward or uncomfortable postures, carrying heavy loads, having disturbed concentration, and working in an unhealthy work environment (Table 3).

**Table 3:** Association between occurrence of workplace injuries (WI) during the period of follow-up and exposures to each physical and each psychosocial risk factors (PSF) among care workers.

		***n***	**%**	**Model**	**95% CI**	**p^c^**
				**predicted rate**		
				**WI^a^^,^ ^b^**		
**Physical risk factors**						
1. Awkward or uncomfortable postures	Low	938	21.2	8.0	4.7–11.2	< 0.001^***^
	Mid	1,724	39.0	22.0	18.1–25.8	
	High	1,756	39.8	28.9	24.7–33.0	
2. Carrying heavy loads	Low	1,715	38.8	13.8	10.7–17.0	< 0.001^***^
	High	2.703	61.2	26.9	23.6–30.2	
3. Vibration or shaking	Low	3,984	90.2	21.5	19.1–24.0	0.122
	High	433	9.8	27.8	19.4–36.1	
4. Loud noise	Low	4,030	91.4	21.6	19.2–24.1	0.169
	High	380	8.6	27.5	18.6–36.4	
5. Concentration	Low	2,611	59.1	18.4	15.6–21.2	< 0.001^***^
	High	1,807	40.9	28.1	23.7–32.4	
6. Unhealthy work environment	Low	2,310	52.3	18.3	15.4–21.3	0.001^**^
	Mid	953	21.6	23.5	17.9–29.1	
	High	1,155	26.1	28.5	23.4–33.6	
Synthetic physical risk score	Low	1,080	24.5	10.2	6.9–13.6	< 0.001^***^
	Mid	1,659	37.5	21.9	18.0–25.8	
	High	1,679	38.0	29.1	24.9–33.4	
**Psychosocial risk factors**						
1. Labor intensity and working time	Low	803	18.2	16.7	11.9–21.4	0.062
	Mid	1,928	43.6	22.2	18.7–25.8	
	High	1,687	38.2	24.9	20.8–29.1	
2. Emotional demand	Low	517	11.7	18.5	12.4–24.7	0.042^*^
	Mid	3,393	76.8	21.5	18.8–24.2	
	High	508	11.5	30.0	22.1–38.0	
3. Autonomy	Low	3,279	74.2	20.0	17.3–22.6	0.017^*^
	Mid	996	22.5	27.0	21.7–32.4	
	High	143	3.4	31.9	17.0–46.8	
4. Social relationships at work	Low	2,389	54.1	16.8	14.0–19.6	< 0.001^***^
	Mid	1,535	34.7	23.5	19.2–27.8	
	High	494	11.2	43.9	34.1–53.7	
5. Conflict of values	Low	2,410	54.5	20.2	17.1–23.2	0.069
	High	2,008	45.5	24.6	20.9–28.3	
6. Job insecurity	Low	588	13.3	17.0	11.1–23.0	< 0.001^***^
	Mid	2,877	65.1	20.5	17.7–23.3	
	High	953	21.6	29.6	23.8–35.4	
Synthetic psychosocial risk score	Low 1^d^	544	12.3	13.2	7.8–18.6	< 0.001^***^
	Low 2^e^	2,761	62.5	19.7	16.9–22.5	
	High	1,113	25.2	31.9	26.3–37.5	

Models predicted rates of WI per 1,000 py and 95% confidence intervals (CI) from Poisson regression analyses with adjustment for covariates. *N* = 4,418.

* *p* < 0.05,

** *p* < 0.01,

*** *p* < 0.001.

a Poisson regression models adjust for gender, age class, educational level, work contract, seniority, sleep problem and use of psychotropic drugs, with an offset (duration of work weighted by part-time work).

b Predicated model rates of WI per 1,000 person-years.

c p-value of incidence rate ratio (global test).

d Low 1 = low PSF exposure.

e Low 2 = low1+ high job insecurity.

We characterized as low, middle and high physical exposure the three groups (synthetic physical score) obtained using the hierarchical classification carried out on the physical factors (Supplementary material 1, second step). The higher this score, the higher the model predicted rate of WI (Table 3). Rates of WI according to gender are presented in Supplementary material 3.

### Psychosocial risk factors and workplace injuries

Poor social relationships at work, job insecurity, lack of autonomy, and emotional demand were statistically associated with the incidence of WI among care workers (Table 3).

Regarding the synthetic PSF risk score, the cluster analysis of the PSF led to the identification of three groups of psychosocial exposure: low PSF exposure, low PSF exposure but high job insecurity, and multiple high PSF exposures (Supplementary material 1, second step). The lowest model predicted rate was for low PSF exposure [low 1: 13.2 WI per 1,000 py (7.8–18.6)]. The rate was higher for low PSF exposure but high job insecurity [low 2: 19.7 WI per 1,000 py (16.9–22.5)]. Finally, the rate was the highest in the case of multiple high exposures to PSF risk factors [31.9 WI per 1,000 py (26.3–37.5)].

Rates of WI according to gender are presented in Supplementary material 4.

### Co-exposures to physical and psychosocial work factors

**Figure 1:**
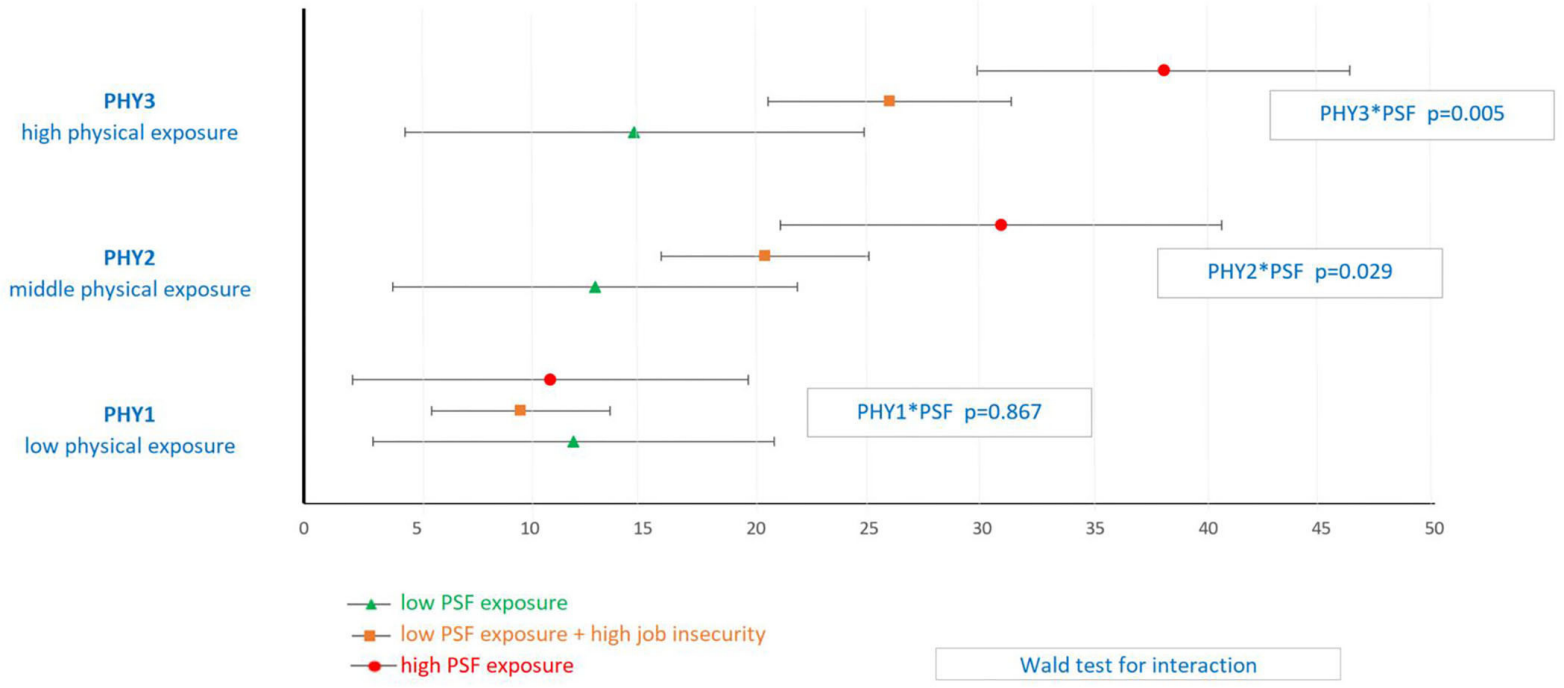
Model predicted rate of workplace injuries for each level of physical (PHY) and psychosocial (PSF) exposure among Care workers^a^. ^a^Poisson regression model with adjustment for covariates: gender, age class, educational level, work contract, seniority, sleep problem and use of psychotropic drugs, with an offset (duration of work weighted by part-time work).

WI were closely related to both the synthetic physical score (*p* < 0.001) and the synthetic PSF risk score (*p* < 0.001) (Figure 1). With low physical exposure, there was no increased risk of WI whatever the level of PSF (*p* = 0.867). Model predicted rates of WI did not differ significantly for the lowest PSF exposed, regardless of the physical exposure. There was a significant increase in predicted injury rates as PSF exposure increased for both middle and high exposure to physical factors (*p* = 0.029 and *p* = 0.005, respectively).

Physical and psychosocial exposures potentiated each other and their co-exposure significantly increased the risk of WI. Model predicted rates of WI per 1,000 py for those most exposed to physical factors was 14.6 WI per 1,000 py [4.5–24.8] when low PSF, 25.9 WI per 1,000 py [20.5–31.3] when low PSF and high job insecurity, and 38.0 [29.8–46.3] when high PSF (*p* < 0.001) (Figure 1).

### Profile of the care workers with the highest rate of WI

Among care workers, 709 workers (16%) were exposed to combinations of physical and psychosocial risk factors with model predicted rates higher than 40 WI per 1,000 py (high-rate group). Their co-exposure consisted mainly of poor social relationships at work and, to a lesser extent, a lack of autonomy, high job insecurity and high labor intensity (Supplementary material 5). Socio-demographic characteristics did not differ between the high-rate group and the other care workers (data not shown).

According to the results of the logistic regression shown in Table 4 (Model 2), work factors that predicted belonging to the high-rate group were as follows: working as nursing assistant or hospital services officer, difficulty of organization in scheduling, overtime, work-family imbalance, and insufficient preventive measures.

**Table 4:** Work activity and work organization characteristics^a^ associated with belonging to care workers exposed to combinations of physical and psychosocial exposures with workplace injuries rates >40 per 1,000 py (*n* = 709).

	**Models 1**			**Model 2**			**Model 3**		
	**Univariate**			**Adjusted for all covariates**			**Model 2 without work activity**		
	**OR**	**95% CI**	***p***	**OR**	**95% CI**	***p***	**OR**	**95% CI**	***p***
**Work activity**									
Nursing assistant			0.013^*^			0.001^**^			
No	Ref			Ref					
Yes	1.27	1.05–1.54		1.43	1.15–1.79		–	–	–
**Hospital services officer**			< 0.001^***^			< 0.001^***^			
No	Ref			Ref					
Yes	1.79	1.41–2.29		2.28	1.72–3.03		–	–	–
**Organization**									
**Schedule forecasting**									
**Modification of schedules by colleagues if unforeseen**			< 0.001^***^			< 0.001^***^			< 0.002^**^
Yes	Ref			Ref			Ref		
No	1.54	1.26–1.87		1.49	1.19–1.85		1.42	1.14–1.76	
**Knowledge of the schedules to be carried out…**			< 0.001^***^			0.005^**^			0.002^**^
At least 1 month in advance	Ref			Ref			Ref		
At least 1 week in advance	1.77	1.41–2.21		1.51	1.18–1.95		1.56	1.21–2.00	
The day before	1.14	0.72–1.80		1.07	0.63–1.84		1.07	0.63–1.84	
**Daily schedules**									
**Type of time control to which one is subjected**			< 0.001^***^			0.011^*^			0.013^*^
No control	Ref			Ref			Ref		
Time switch, badge/signature, time card and related	1.39	1.10–1.76		1.33	1.03–1.71		1.32	1.02–1.69	
Control by management or others, e.g., colleagues	1.38	1.15–1.65		1.30	1.07–1.60		1.30	1.07–1.59	
**Overtime, on–call, vacation**									
**Working beyond the scheduled time**			< 0.001^***^			< 0.001^***^			0.026^*^
Never	Ref			Ref			Ref		
Sometimes	1.27	0.99–1.61		1.26	0.97–1.65		1.18	0.90–1.54	
Often	1.78	1.37–2.31		1.81	1.34–2.45		1.50	1.12–2.01	
Every day	1.71	1.20–2.43		1.85	1.22–2.81		1.46	0.97–2.19	
**On-calls**			0.044^*^			0.044^*^			0.100
Never	Ref			Ref			Ref		
Sometimes	1.19	0.93–1.52		1.36	1.02–1.80		1.24	0.94–1.63	
Usually	1.36	0.88–2.11		1.43	0.89–2.30		1.48	0.93–2.37	
**In case of unforeseen personal or family events, to be absent from work for even a few hours is**			< 0.001^***^			< 0.001^***^			< 0.001^***^
Easy	Ref			Ref			Ref		
Possible but not easy	1.82	1.50–2.19		1.52	1.23–1.87		1.54	1.25–1.89	
Impossible	1.92	1.54–2.40		1.61	1.25–2.08		1.65	1.28–2.12	
**Prevention**									
**Provision of individual protective equipment by the employer (gloves, glasses, safety shoes, harness…)**			< 0.001^***^			< 0.001^***^			< 0.001^***^
Yes	Ref			Ref			Ref		
No	2.65	2.10–3.34		2.23	1.72–2.89		2.31	1.79–3.00	
**Written instructions available to preserve safety or health in the workplace (except evacuation instructions in case of fire)**			< 0.001^***^			< 0.001^***^			< 0.001^***^
Yes	Ref			Ref			Ref		
Yes but not applicable	1.69	1.34–2.13		1.50	1.76–1.92		1.53	1.20–1.95	
No	1.37	1.13–1.65		1.64	1.33–2.02		1.53	1.25–1.88	

Odds Ratio (OR) and 95% confidence intervals (CI) from logistic regression analyses. *N* = 4,418.

* *p* < 0.05,

** *p* < 0.01,

*** *p* < 0.001.

a Logistic regression models after stepwise selection on all covariates describing sociodemographic characteristics, work activity and work organization.

The area under the curve was 0.67. Finally, the higher the probability of belonging to the high-rate group, the higher the risk of WI [OR = 1.05 (1.00–1.09) with *p* = 0.031].

## Discussion

Among the 4,418 care workers, workplace injuries were closely linked to both reported physical and psychosocial exposures. Physical and PSF exposures potentiated each other and their co-exposure increased the risk of occurrence of WI. The highest rate of predicted rate of WI concerned care workers with the highest co-exposure to psychosocial and physical factors: 38 WI per 1,000 py. Moreover, with low exposure to physical risk factors, there was no increased risk of the occurrence of WI, whatever the level of PSF exposure. Similarly, with low PSF exposure, the predicted rates of WI were not different according to the level of exposure to physical factors.

These results are in line with the findings of our previous study on a population of workers from all sectors of activity: the risk of WI was highest among workers with high physical exposures regardless of the psychosocial exposures. Indeed, the difference in rates of WI between high and low PSF exposures seemed to increase with increasing physical exposure, but not significantly (25).

When focusing on the high WI rate group, the PSF mainly involved consisted of poor social relationships at work, whatever the type of physical factors and, to a lesser extent, low autonomy, high job insecurity and high labor intensity. Our results are consistent with the literature. Studies found that nursing assistant WI rates were higher in case of low supervisor support (28), nurses who reported higher social support from co-workers had lower WI rates (29) and workers in the health and welfare sector exposed to violence and harassment from supervisors, colleagues or others were associated with a high WI rate (20). In this population, low autonomy was also associated with WI (20) as well as high workload (30, 31) and precarious employment resulting in high job insecurity (32).

We identified two main types of occupations in our high-rate group: nursing assistants and hospital services officer. Similarly, higher WI rates among nursing assistants, relative to nurses, were previously reported in 4 studies (24, 33–35). Moreover, emergency medical technicians and paramedics had a WI rate about three times the United States average for all occupations (1). Among hospital workers, emergency medical technicians had the highest rate of WI (33). A recent Australian study provided evidence of a high WI rate among health and social care workers. However, they found the highest rates of WI for social workers and ambulance officers, which was not the case in our study (3). Contrary to nursing assistants and hospital services officers, being a nurse was not associated with exposures leading to the highest WI rates. A previous study, focusing on the risk perception of musculoskeletal injury, found that most critical care nurses were concerned about their ergonomic job risks (36), which may be one explanation for lowest WI rate that we observed in nurses. Moreover, Rodriguez-Acosta et al. (34) observed that most of the WI for health care workers were related to the process of delivering direct patient care, nurses performing fewer tasks compared to assistant nurses and having a lower risk of lifting injuries than assistant nurses.

We identified some organizational factors that predicted belonging to the high-rate group. These care workers had in common: schedule inflexibility, work duration, mandatory overtime, impossibility or difficulty to be absent in the case of unforeseen personal or family events and inadequate safety prevention policy (i.e., provision of individual protective equipment and availability of written instructions). In the literature on nursing assistants, the frequency of working mandatory overtime (37), as well as not having sufficient time to complete patient activities of daily living (37, 38), were strongly associated with the occurrence of WI. The absence or unavailability of mechanical lifting equipment when needed increased the risk of WI among nursing assistants (33, 38). When nursing assistants declared available facilities training to reduce injuries, the risk of WI was lower (38). Finally, nurses who reported better safety leadership, greater safety diligence or better ergonomic practices, had lower WI rates (29).

Our results were in line with previous literature on most of the factors known to influence the occurrence of WI. WI rates were statistically higher among the youngest workers (39–41), those with a lower educational level (39, 41), subject to precarious contracts (42), or with poor seniority (43), sleep problems (44) or using psychotropic drugs (45). While the literature highlighted higher rates of WI among men than women in other sectors of activity (39, 40), the rate of WI among care workers was highest for women in our study. These results were in line with other studies (1, 12). Within the same occupations, men were globally more likely to be exposed to physical hazards compared to women, but women in healthcare occupations were more exposed to prolonged standing, kneeling, lifting, and other material handling than men, increasing the likelihood of WI (46).

To the best of our knowledge, this is the first epidemiological study to analyse the impact of multiple exposure to physical and psychosocial factors on the occurrence of WI among care workers. The most striking result in our study is the significant interaction between physical and PSF exposures in the occurrence of WI among care workers. Moreover, no potentiation of exposures was observed in either low physical or low PSF exposure. In comparison to the care workers, model predicted rates of WI for all workers were not significantly different between two low PSF exposure categories (data not shown). More specifically, same analyses in blue collar workers seems to report different patterns of interaction between physical factors and PSF (data not shown). The levels of exposure to physical factors, mainly awkward, uncomfortable postures and carrying heavy loads, were high among both blue collar and care workers. Both had high levels of psychosocial exposure but blue-collar workers reported low autonomy, lack of reward and low job insecurity more frequently, while care workers mainly reported high emotional demand, poor social support, high conflicts of values, excessive workload and work complexity (data not shown). These results lead us to assume that the potentiation of physical and psychosocial factors differs according to the type of FPS and should be investigated in depth.

Our study have several strengths. This survey covered workers who were representative of the French working population. The large number of participants allowed us to focus on specific occupational sectors. Matching with the databases of the National Health Insurance Funds' compensation system provided independent and systematic data on WI with detailed information on the nature, circumstances and duration of any sick leave. Moreover, the detailed questionnaires obtained before the incidence of WI allowed the accurate characterization of physical and psychosocial exposures, eliminating any information bias inherent to retrospective information collection.

In order to estimate potential selection bias, we compared our sample with those lost to follow-up (not interviewed in 2016) and those excluded due to absence of national insurance ID. Globally, there were no differences between these groups in terms of overall health and reported number of WI in the year before 2013.

A final issue might be underreporting of WI to the employer in small companies due to the lack of a reference person to pass on the information or the difficulty/impossibility of replacement when absent (47). Regarding our care workers, the raw WI rate was higher in small companies than in the largest companies. Therefore, underreporting in small companies does not seem to be a major issue in our study.

## Conclusion

Our findings highlight the need to take into account psychosocial factors in addition of only considering physical factors when analyzing WI occurrence, as usually done.

From a theoretical perspective, future research should focus on identifying the most deleterious combinations of risks from multiple exposures to physical and psychosocial risk factors in the occurrence of occupational injuries.

These results provide practical keys points for the prevention of WI among care workers. Prevention actions must be taken to reduce both physical and psychosocial exposure. More specifically, interventions targeted at reducing the handling of loads or people and promoting an organization more centered on the management of human resources could jointly reduce the physical and psychosocial risks that cause many WI in the field of care.

## Data availability statement

The original contributions presented in the study are included in the article/Supplementary material, further inquiries can be directed to the corresponding author/s.

## Ethics statement

All the subjects gave their free and informed consent for participation in the Working Conditions survey. Access to certain confidential data for this work was made possible within a secure environment offered by CASD—Centre d'accès sécurisé aux données (Ref. 10.34724/CASD). Our study was performed in accordance with the ethical standards in force and received the necessary regulatory approvals (Visa Comité du secret statistique-ME463 and CNIL-2215533).

## Author contributions

RC designed the study, reviewed the literature, performed data management and statistical analyses, and drafted the manuscript. SB designed the study, reviewed the literature, oversaw statistical analyses, and drafted the manuscript. PW participated in study design, oversaw statistical analyses, and drafted the manuscript. CP participated in study design and drafted the manuscript. All authors collaborated interactively, read, and approved the final manuscript.
