# Supplementary material for: Co-exposures to physical and psychosocial work factors increase the occurrence of workplace injuries among French care workers
Source: Front Public Health. 2022 Dec 13;10:1055846. doi: 10.3389/fpubh.2022.1055846 (PMC9792696; doi:10.3389/fpubh.2022.1055846)
Supplement: Supplementary file 1 [file Data_Sheet_1.PDF]

Supplemental Material 1. Characterisation of physical and psychosocial exposures.

We characterized the co-exposures to physical and psychosocial factors for the 17 831 participants.

1. FIRST STEP

The physical and psychosocial items were mainly dichotomous or coded on a 4-point Likert scale. Given the large number of questions, we grouped them into broad categories or subgroups of physical factors and psychosocial factors identified by a group of French experts.

When the category or subgroup was represented by (1) only 1 item, the score took the value of the item; (2) 2 items, the score corresponded to their crossing; (3) more than 2 items, we performed Multiple Correspondence Analysis (MCA) on imputed data associated with an Ascending Hierarchical Classification (AHC). We fixed the output to three clusters. Each cluster was described according to the mean of the value of the different items composing it (standardised from 0 to 1), in order to rank the clusters (from 1, the lowest, to 3, the highest exposure). When no ranking was possible or when the size was insufficient, we performed another AHC, choosing to create two clusters rather than three.

green	yellow	red
lowest mean value	middle mean value	highest mean value

a. PHYSICAL risk factors (PHY)

PHY_score_fam1 - Awkward and uncomfortable postures (4 items)

PHY_score_fam1	N	%	PHY1_Cwdebou	PHY1_Cwpostu	PHY1_Cwmvt	PHY1_Cwdepla
1	6 975	39,12	0,00	0,12	0,06	0,00
2	5 428	30,44	0,83	0,00	0,39	0,60
3	5 428	30,44	0,88	1,00	0,82	0,60

PHY1_Cwdebou Does your job require you to stand for long periods of time?
PHY1_Cwpostu Does the performance of your job require you to stay in another awkward or tiring posture for a long time?
PHY1_Cwmvt Does your job require you to perform painful or tiring movements?

PHY1_Cwdepla Does the performance of your job require you to make long or frequent trips on foot?

PHY_score_fam2 - Carrying heavy loads (1 item)

Does your job require you to carry or move heavy loads?

PHY_score_fam2		
	N	%
0	10 168	57,02
1	7 658	42,95

PHY_score_fam3 - Vibration or shaking (1 item)

Does your job cause you to shake or vibrate?

PHY_score_fam3		
	N	%
0	15 111	84,75
1	2 717	15,24

PHY_score_fam4 – Loud noise (1 item)

Can you hear a person placed 2 or 3 meters away from you when they speak to you ?

PHY_score_fam4			
		N	%
	0	14 908	83,61
	1	2 878	16,14
	.	45	0,25

PHY_score_fam5 - Concentration (4 items)

PHY_score_fam5						
	N	%	PHY5_Cwvue	PHY5_Cwletr	PHY5_Cwminus	PHY5_Cwviso
0	11 723	65,75	0,29	0,27	0,00	0,00
1	6 108	34,25	0,62	0,56	0,59	0,71

PHY5_Cwvue	Does your job require you to keep your eyes on your work?
PHY5_Cwletr	Does your job require you to read small, poorly printed, poorly written letters or numbers?
PHY5_Cwminus	Does your work require you to read and examine very small objects, small details?
PHY5_Cwvisio	Does the performance of your job require you to read and pay attention to brief, unpredictable, or hard-to-detect visual or audible signals?

PHY_score_fam6 – Unhealthy work environment (9 items)

PHY_score_fam6	N	%	PHY6_Hygsal	PHY6_Hygchumi	PHY6_Hygcour	PHY6_Hygodeur	PHY6_Hygchaud
1	8 776	49,22	0,06	0,02	0,09	0,12	0,12
2	3 722	20,87	0,13	0,05	0,27	0,26	0,27
3	5 333	29,91	0,74	0,63	0,83	0,74	0,72
			PHY6_Hygfroi(	PHY6_Hygsani	PHY6_Hygvue	PHY6_Hygpriv	
		1	0,10	0,02	0,00	0,00	
		2	0,30	0,07	0,56	0,66	
		3	0,77	0,35	0,20	0,20	

Does your job or workplace have the following disadvantages?

PHY6_Hygsal	dirt
PHY6_Hygchumi	humidity
PHY6_Hygcour	draughts
PHY6_Hygodeur	bad smells
PHY6_Hygchaud	high temperature
PHY6_Hygfroid	low temperature
PHY6_Hygsani	lack or poor condition of sanitary facilities
PHY6_Hygvue	no view on the external environment
PHY6_Hygpriv	lack of privacy

b. PSYCHOSOCIAL risk factors

RPS_score_fam1: Labour intensity and working time

RPS_score_fam1	N	%	RPS_score_ssfam1_1	RPS_score_ssfam1_2	RPS_score_ssfam1_3
1	5 146	28,86	0,20	0,35	0,00
2	6 706	37,61	0,23	0,44	0,50
3	5 979	33,53	0,36	0,63	1,00

RPS_SCORE_SSFAM1_1 Excessive workload
RPS_SCORE_SSFAM1_2 Time pressure
RPS_SCORE_SSFAM1_3 Work Complexity

RPS_score_fam2: Emotional demand

RPS_score_fam2	N	%	RPS_score_ssfam2_1	RPS_score_ssfam2_2	RPS_score_ssfam2_3	RPS_score_ssfam2_4
1	3 404	19,09	0,50	0,16	0,21	0,02
2	12 949	72,62	0,56	0,20	0,23	0,02
3	1 478	8,29	0,77	0,59	0,52	0,38

RPS_SCORE_SSFAM2_1 Contact with suffering
RPS_SCORE_SSFAM2_2 Relationship with the public (external violence)
RPS_SCORE_SSFAM2_3 Emotional dissonance
RPS_SCORE_SSFAM2_4 Fear for safety during work

RPS_score_fam3: Lack of autonomy

RPS_score_fam3	N	%	RPS_score_ssfam3_1	RPS_score_ssfam3_2	RPS_score_ssfam3_3	RPS_score_ssfam3_4	RPS_score_ssfam3_5
1	12 459	69,87	0,01	0,16	0,21	0,42	0,26
2	4 419	24,78	0,35	0,43	0,69	0,34	0,44
3	953	5,34	0,77	0,70	0,82	0,38	0,48

RPS_SCORE_SSFAM3_1 Monotony and boredom
RPS_SCORE_SSFAM3_2 Lack of pleasure at work
RPS_SCORE_SSFAM3_3 Skills not fully utilized or developed
RPS_SCORE_SSFAM3_4 Unpredictability
RPS_SCORE_SSFAM3_5 No choice of how a job is done

RPS_score_fam4: Social relationships at work

RPS_score_fam4	N	%	RPS_score_ssfam4_1	RPS_score_ssfam4_2	RPS_score_ssfam4_3	RPS_score_ssfam4_4	RPS_score_ssfam4_5
1	9 931	55,70	0,02	0,15	0,20	0,18	0,02
2	5 825	32,70	0,16	0,35	0,48	0,49	0,17
3	2 075	11,60	0,41	0,43	0,54	0,78	0,46
			RPS_score_ssfam4_6	RPS_score_ssfam4_7	RPS_score_ssfam4_8	RPS_score_ssfam4_9	RPS_score_ssfam4_10
			0,05	0,02	0,14	0,50	0,18
			0,28	0,28	0,48	0,74	0,44
			0,68	0,71	0,82	0,82	0,73

RPS_SCORE_SSFAM4_1	Internal violence
RPS_SCORE_SSFAM4_2	Poor relationships with colleagues (team cooperation and integration in a team)
RPS_SCORE_SSFAM4_3	Poor relationships with colleagues (team autonomy, engagement)
RPS_SCORE_SSFAM4_4	Poor relationship with the hierarchy (support from superiors)
RPS_SCORE_SSFAM4_5	Poor relationship with the hierarchy (leadership)
RPS_SCORE_SSFAM4_6	Poor relationship with the hierarchy (organisational justice)
RPS_SCORE_SSFAM4_7	Lack of reward
RPS_SCORE_SSFAM4_8	Lack of career prospects
RPS_SCORE_SSFAM4_9	Insufficient salary
RPS_SCORE_SSFAM4_10	Inadequate social recognition of the job

RPS_score_fam5: Conflict of values

RPS_score_fam5	N	%	RPS_score_ssfam5_1	RPS_score_ssfam5_2
0	10 763	60,36	0,00	0,00
1	6 884	38,61	0,24	0,82

RPS_SCORE_SSFAM5_1	Ethical conflicts
RPS_SCORE_SSFAM5_2	No opportunity to perform high-quality jobs

RPS_score_fam6: Job insecurity

RPS_score_fam6	N	%	RPS_score_ssfam6_1	RPS_score_ssfam6_2	RPS_score_ssfam6_3
1	2 257	12,66	0,00	0,26	0,13
2	11 304	63,40	0,50	0,34	0,10
3	427	23,95	0,93	0,44	0,35

RPS_SCORE_SSFAM6_1	Job instability
RPS_SCORE_SSFAM6_2	Lack of work sustainability
RPS_SCORE_SSFAM6_3	Occupational changes

2. SECOND STEP

We performed Multiple Correspondence Analysis (MCA) on the previous categories defined at first step with an Ascending Hierarchical Classification (AHC). We fixed the output to three clusters. Each cluster was described according to the mean value of the different categories composing it (standardised from 0 to 1), in order to rank the clusters (from 1, the lowest, to 3, the highest exposure). When no ranking was possible or when the size was insufficient, we performed another AHC, choosing to create two clusters rather than three.

a. PHY_synth – Physical risk factor exposure score (6 categories)

PHY_synth	N	%	PHY_score_fam1	PHY_score_fam2	PHY_score_fam3	PHY_score_fam4	PHY_score_fam5	PHY_score_fam6
1	7 160	40,15	0,08	0,08	0,03	0,06	0,16	0,17
2	5 013	28,11	0,50	0,48	0,05	0,15	0,36	0,37
3	5 658	31,73	0,89	0,83	0,40	0,30	0,56	0,72

1 => low physical exposure

2=> middle physical exposure

3=> high physical exposure

b. RPS_synth: Psychosocial Risk Factors exposure (6 categories)

RPS_synth	N	%	RPS_score_fam1	RPS_score_fam2	RPS_score_fam3
1	2 146	12,00	0,40	0,44	0,14
2	11 479	64,40	0,47	0,40	0,11
3	4 206	23,60	0,73	0,57	0,37
			RPS_score_fam4	RPS_score_fam5	RPS_score_fam6
		1	0,12	0,14	0,05
		2	0,16	0,13	0,57
		3	0,67	0,44	0,77

1=> low1: low PSF exposure

2=> low2: low PSF exposure + high job insecurity

3=> high PSF exposure
