# Supplementary material for: Co-exposures to physical and psychosocial work factors increase the occurrence of workplace injuries among French care workers
Source: Front Public Health. 2022 Dec 13;10:1055846. doi: 10.3389/fpubh.2022.1055846 (PMC9792696; doi:10.3389/fpubh.2022.1055846)
Supplement: Supplementary file 2 [file Data_Sheet_2.PDF]

Supplemental Material 2. Models predicted rates of workplace injuries per 1 000 py among care workers (n= 4 418)

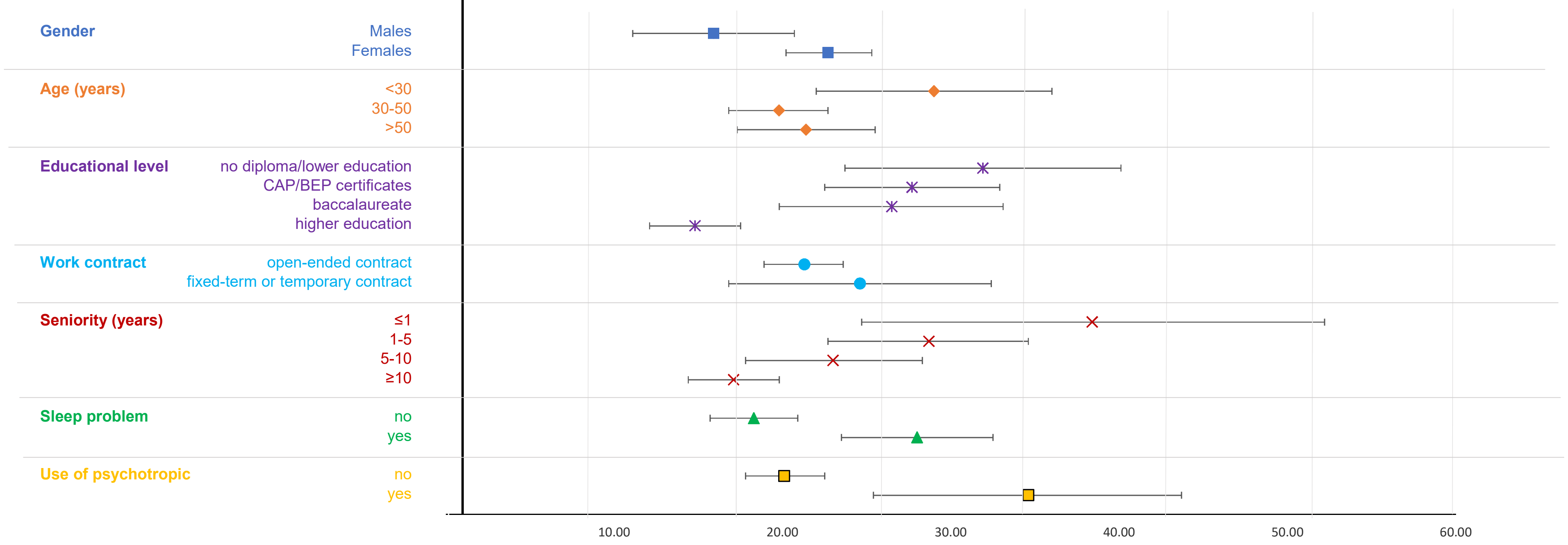

Poisson regression models for each adjustment variables selected after a stepwise selection (sociodemographics, health, activity) on care workers
