# Supplementary material for: Co-exposures to physical and psychosocial work factors increase the occurrence of workplace injuries among French care workers
Source: Front Public Health. 2022 Dec 13;10:1055846. doi: 10.3389/fpubh.2022.1055846 (PMC9792696; doi:10.3389/fpubh.2022.1055846)
Supplement: Supplementary file 3 [file Data_Sheet_3.PDF]

Supplemental material 3. Association between occurrence of workplace injuries (WI) during the period of follow-up and exposures to each physical risk factors among care workers, by gender. Models predicted rates of worplaces injuries per 1000 py and 95% confidence intervals (CI) from Poisson regression analyses with adjustement for covariates. N=4 418

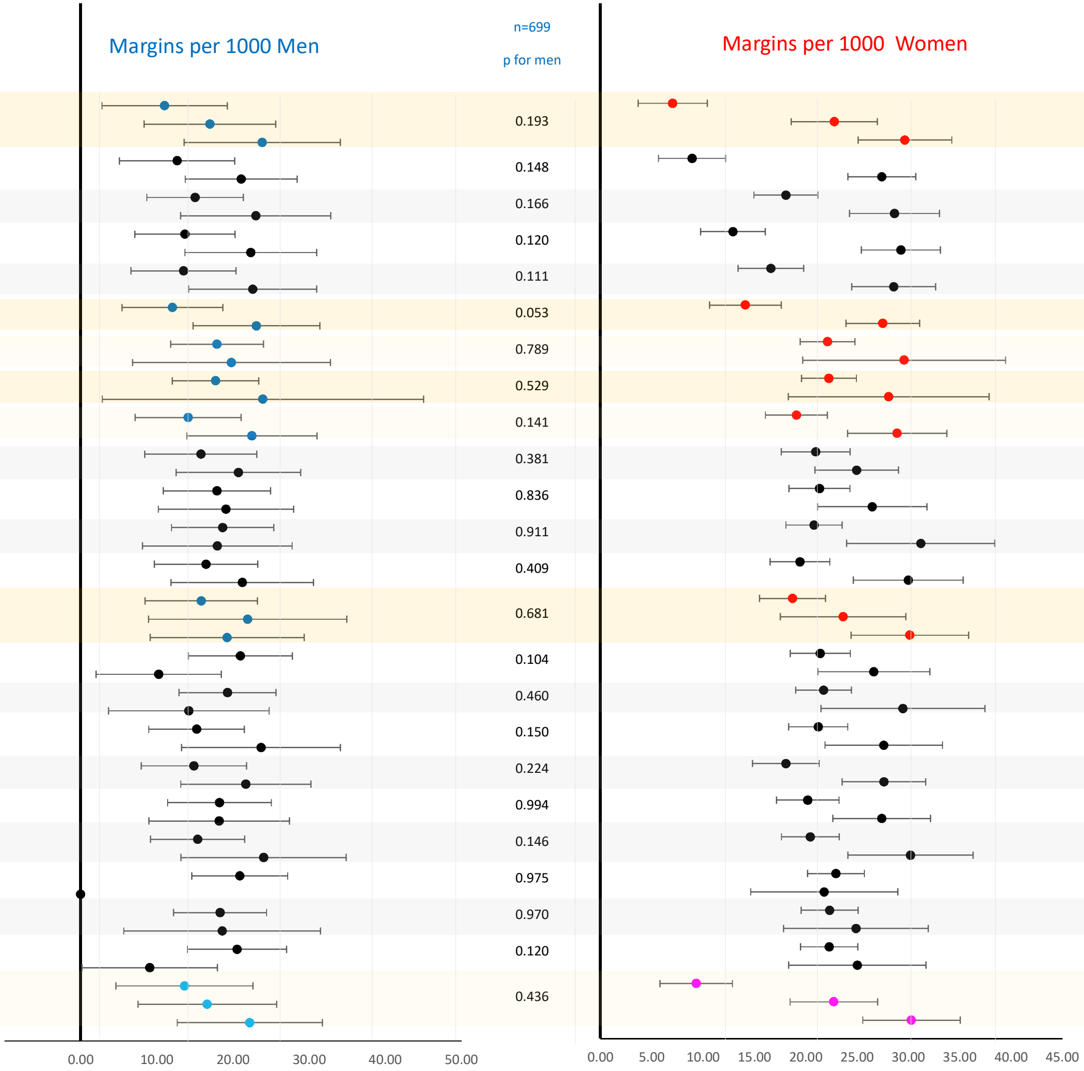

Risk factor	Level	n=699<br>p for men	n=3719<br>[illegible] for women	p women vs men
**1. Awkward or uncomfortable postures**	low / mid / high	0.193	**<0.001*** **	0.345
standing for long periods of time	low / high	0.148	**<0.001*** **	0.127
staying in another awkward or tiring posture for a long time	low / high	0.166	**<0.001*** **	0.914
performing painful or tiring movements	low / high	0.120	**<0.001*** **	0.337
making long or frequent trips on foot	low / high	0.111	**<0.001*** **	0.932
**2. Carrying heavy loads**	low / high	0.053	**<0.001*** **	0.962
**3. Vibration or shaking**	low / high	0.789	0.109	0.649
**4. Loud noise**	low / high	0.529	0.217	0.893
**5. Concentration**	low / high	0.141	**<0.001*** **	0.881
keeping the eyes on the work	low / high	0.381	0.136	0.768
reading small, poorly printed, poorly written letters or numbers	low / high	0.836	0.087	0.649
examining very small objects, small details	low / high	0.911	**0.003*** *	0.217
reading and paying attention to brief, unpredictable, or hard-to-detect visual or audible signals	low / high	0.409	**<0.001*** **	0.584
**6. Unhealthy work environment**	low / mid / high	0.681	**0.001*** **	0.645
dirt	low / high	0.104	0.084	**0.042***
humidity	low / high	0.460	**0.046***	0.166
draughts	low / high	0.150	**0.037***	0.589
bad smells	low / high	0.224	**<0.001*** **	0.889
high temperature	low / high	0.994	**0.010***	0.367
low temperature	low / high	0.146	**0.002*** *	0.862
lack or poor condition of sanitary facilities	low / high	0.975	0.776	0.975
no view of the external environment	low / high	0.970	0.494	0.825
lack of privacy	low / high	0.120	0.440	0.088
**Physical risk factors exposure**	low / mid / high	0.436	**<0.001*** **	0.270

Poisson regression models adjust for gender, age class, educational level, work contract, seniority, sleep problem and use of psychotropic drugs, with an offset (duration of work weighted by part-time work).
