# Supplementary material for: Co-exposures to physical and psychosocial work factors increase the occurrence of workplace injuries among French care workers
Source: Front Public Health. 2022 Dec 13;10:1055846. doi: 10.3389/fpubh.2022.1055846 (PMC9792696; doi:10.3389/fpubh.2022.1055846)
Supplement: Supplementary file 4 [file Data_Sheet_4.PDF]

**Supplemental material 4.** Association between occurrence of workplace injuries (WI) during the period of follow-up and exposures to each psychosocial risk factors (PSF) among care workers, by gender. Models predicted rates of worplaces injuries per 1000 py and 95% confidence intervals (CI) from Poisson regression analyses with adjustement for covariates. N=4 418

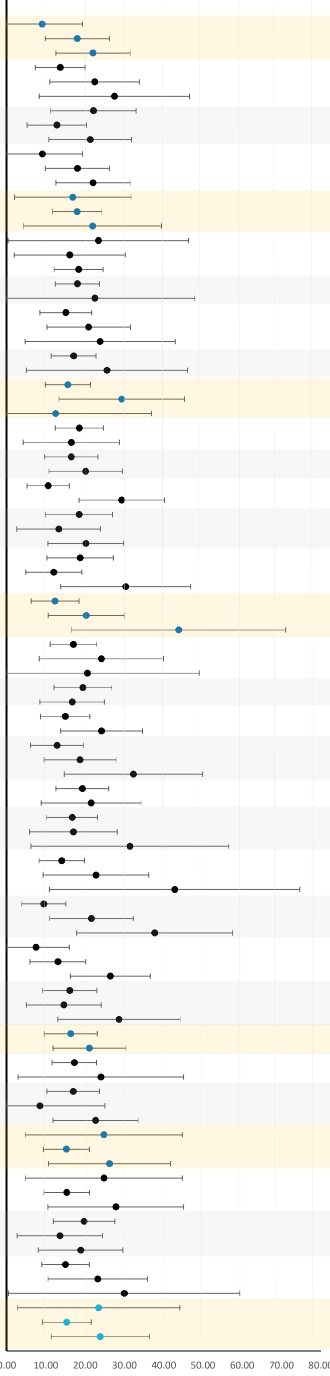


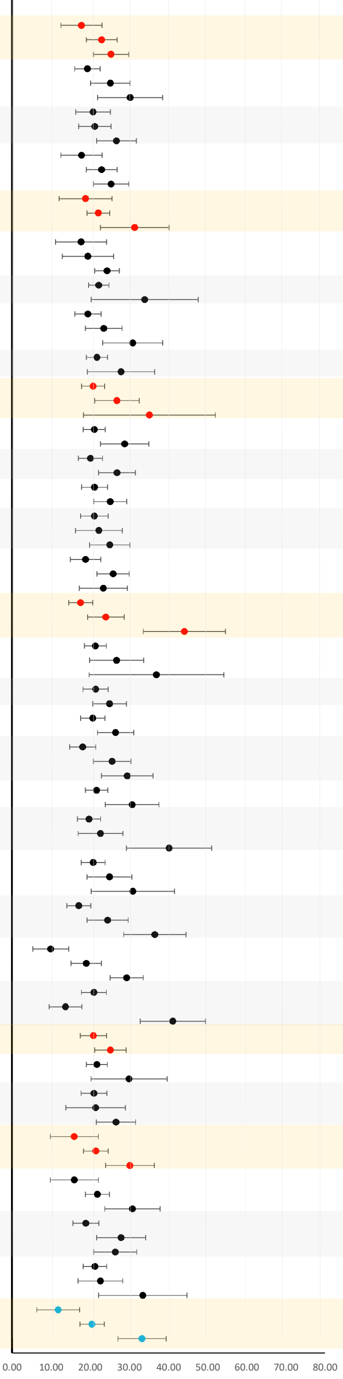


| Psychosocial risk factor | Levels | n=699 p for men | n=3719 p for women | p women vs men |
|---|---|---|---|---|
| dimension 1: Labour intensity and working time | 1, 2, 3 | 0.343 | 0.139 | 0.709 |
| 1.1 Excessive workload | 1, 2, 3 | 0.180 | 0.014* | 0.779 |
| 1.2 Time pressure | 1, 2, 3 | 0.319 | 0.149 | 0.396 |
| 1.3 Work Complexity | 1, 2, 3 | 0.343 | 0.139 | 0.709 |
| dimension 2: Emotional demand | 1, 2, 3 | 0.888 | 0.043* | 0.923 |
| 2.1 Contact with suffering | 1, 2, 3 | 0.852 | 0.174 | 0.609 |
| 2.2 Poor relationship with the public | 1, 2 | 0.713 | 0.047* | 0.747 |
| 2.3 Emotional dissonance | 1, 2, 3 | 0.493 | 0.011** | 0.925 |
| 2.4 Fear for safety during work | 0, 1 | 0.361 | 0.142 | 0.743 |
| dimension 3: Lack of autonomy | 1, 2, 3 | 0.158 | 0.033* | 0.413 |
| 3.1 Monotony and boredom | 0, 1 | 0.777 | 0.016* | 0.319 |
| 3.2 Lack of pleasure at work | 0, 1 | 0.523 | 0.015* | 0.789 |
| 3.3 Skills not fully utilized or developed | 0, 1 | 0.002** | 0.139 | 0.013** |
| 3.4 Unpredictability | 1, 2, 3 | 0.677 | 0.455 | 0.745 |
| 3.5 No choice of how a job is done | 1, 2, 3 | 0.077 | 0.057 | 0.050 |
| dimension 4: Social relationships at work | 1, 2, 3 | 0.008** | <0.001*** | 0.717 |
| 4.1 Internal violence | 1, 2, 3 | 0.645 | 0.039* | 0.841 |
| 4.2 Poor relationships with colleagues (cooperation and integration in a team) | 0, 1 | 0.631 | 0.187 | 0.363 |
| 4.3 Poor relationships with colleagues (team autonomy and work engagement) | 0, 1 | 0.115 | 0.036* | 0.476 |
| 4.4 Poor relationship with the hierarchy (support from superiors) | 1, 2, 3 | 0.057 | 0.002** | 0.504 |
| 4.5 Poor relationship with the hierarchy (leadership) | 0, 1 | 0.755 | 0.008** | 0.518 |
| 4.6 Poor relationship with the hierarchy (organisational justice) | 1, 2, 3 | 0.366 | <0.001*** | 0.953 |
| 4.7 Lack of reward | 1, 2, 3 | 0.030* | 0.080 | 0.296 |
| 4.8 Lack of career prospects | 1, 2, 3 | 0.003** | <0.001*** | 0.326 |
| 4.9 Insufficient salary | 1, 2, 3 | 0.027* | <0.001*** | 0.752 |
| 4.10 Inadequate social recognition of the job | 1, 2, 3 | 0.186 | <0.001*** | 0.655 |
| dimension 5: Conflict of values | 0, 1 | 0.406 | 0.103 | 0.843 |
| 5.1 Ethical conflicts | 0, 1 | 0.490 | 0.070 | 0.985 |
| 5.2 No opportunity to perform high-quality jobs | 1, 2, 3 | 0.475 | 0.156 | 0.765 |
| dimension 6: Job insecurity | 1, 2, 3 | 0.246 | 0.005** | 0.289 |
| 6.1 Job instability | 1, 2, 3 | 0.211 | 0.007** | 0.264 |
| 6.2 Lack of work sustainability | 1, 2, 3 | 0.714 | 0.012** | 0.232 |
| 6.3 Occupational changes | 1, 2, 3 | 0.267 | 0.053 | 0.595 |
| cluster Psychosocial Risk Factors exposure | 1, 2, 3 | 0.364 | <0.001*** | 0.179 |

Poisson regression models adjust for gender, age class, educational level, work contract, seniority, sleep problem and use of psychotropic drugs, with an offset (duration of work weighted by part-time work).
