# Supplementary material for: Co-exposures to physical and psychosocial work factors increase the occurrence of workplace injuries among French care workers
Source: Front Public Health. 2022 Dec 13;10:1055846. doi: 10.3389/fpubh.2022.1055846 (PMC9792696; doi:10.3389/fpubh.2022.1055846)

Supplementary Material 5. Model predicted rates of worplace injuries per 1000 py for each pairwise combination of physical and psychosocial risks exposure among Care workers*.

PHY / PSF		1. Labour intensity and working time			2. Emotional demand			3. Lack of autonomy			4. Social relationships at work			5. Conflict of values		6. Job insecurity		
		low	mid	high	low	mid	high	low	mid	high	low	mid	high	low	high	low	mid	high
1. Awkward or uncomfortable postures	low	6.88	7.41	10.25	10.59	7.39	8.32	7.27	11.69	0.00	5.87	9.09	23.18	8.59	6.76	8.50	6.41	13.25
	mid	18.62	19.40	26.86	24.14	20.32	31.60	19.91	27.21	33.87	19.42	21.66	38.86	20.34	24.14	19.12	19.93	30.39
	high	24.44	31.99	27.20	18.73	29.04	34.31	26.99	31.82	38.52	21.09	29.34	51.73	27.26	30.28	20.38	28.00	34.00
2. Carrying heavy loads	low	11.19	12.39	19.55	19.03	12.58	16.33	12.79	15.68	26.99	11.77	14.24	29.85	13.54	14.38	11.62	11.80	22.56
	high	24.51	27.68	26.82	18.34	26.66	34.42	24.41	32.35	35.12	20.47	27.61	48.74	24.92	28.87	21.30	25.51	32.68
3. Vibration or shaking	low	16.02	20.45	25.96	18.77	20.93	29.29	19.48	26.17	32.37	16.96	23.37	40.76	19.20	24.47	16.80	20.20	28.04
	high	25.00	41.29	18.71	15.68	27.35	33.02	25.11	33.22	30.14	14.73	24.47	58.90	30.24	25.41	19.41	23.54	40.69
4. Loud noise	low	16.50	21.56	24.47	17.83	20.95	30.15	19.56	26.58	30.03	16.13	23.49	44.12	19.96	23.71	15.06	20.30	29.08
	high	19.03	31.35	27.62	24.81	27.54	29.96	25.42	28.81	41.42	24.86	24.13	42.94	23.04	31.73	39.07	23.25	32.09
5. Concentration	low	13.69	19.48	20.79	15.45	18.18	26.25	16.13	23.00	31.36	15.35	20.11	33.88	16.89	20.59	14.27	16.54	26.89
	high	29.40	26.82	29.25	28.95	26.84	33.14	25.96	32.95	32.47	19.73	27.88	52.73	26.33	29.71	22.54	26.83	32.95
6. Unhealthy work environment	low	15.63	17.59	21.68	14.24	18.40	25.44	17.21	21.02	28.63	15.57	20.73	35.46	17.51	19.74	10.77	18.40	24.26
	mid	7.87	24.94	27.18	31.43	22.23	25.24	20.57	28.90	43.58	16.96	25.49	38.66	19.48	27.52	23.72	20.62	30.92
	high	25.76	31.19	27.21	22.34	27.27	36.48	25.93	33.68	30.99	20.47	25.33	52.53	28.48	28.51	31.58	24.94	35.20

* adjusted for gender, age class, level of education, work contract, seniority, sleep problem and use of psychotropic drugs

model predicted rate greater than 40 WI per 1000 person-years

Model predicted rates of worplace injuries for each pairwise combination of physical and psychosocial risks exposure among Care workers

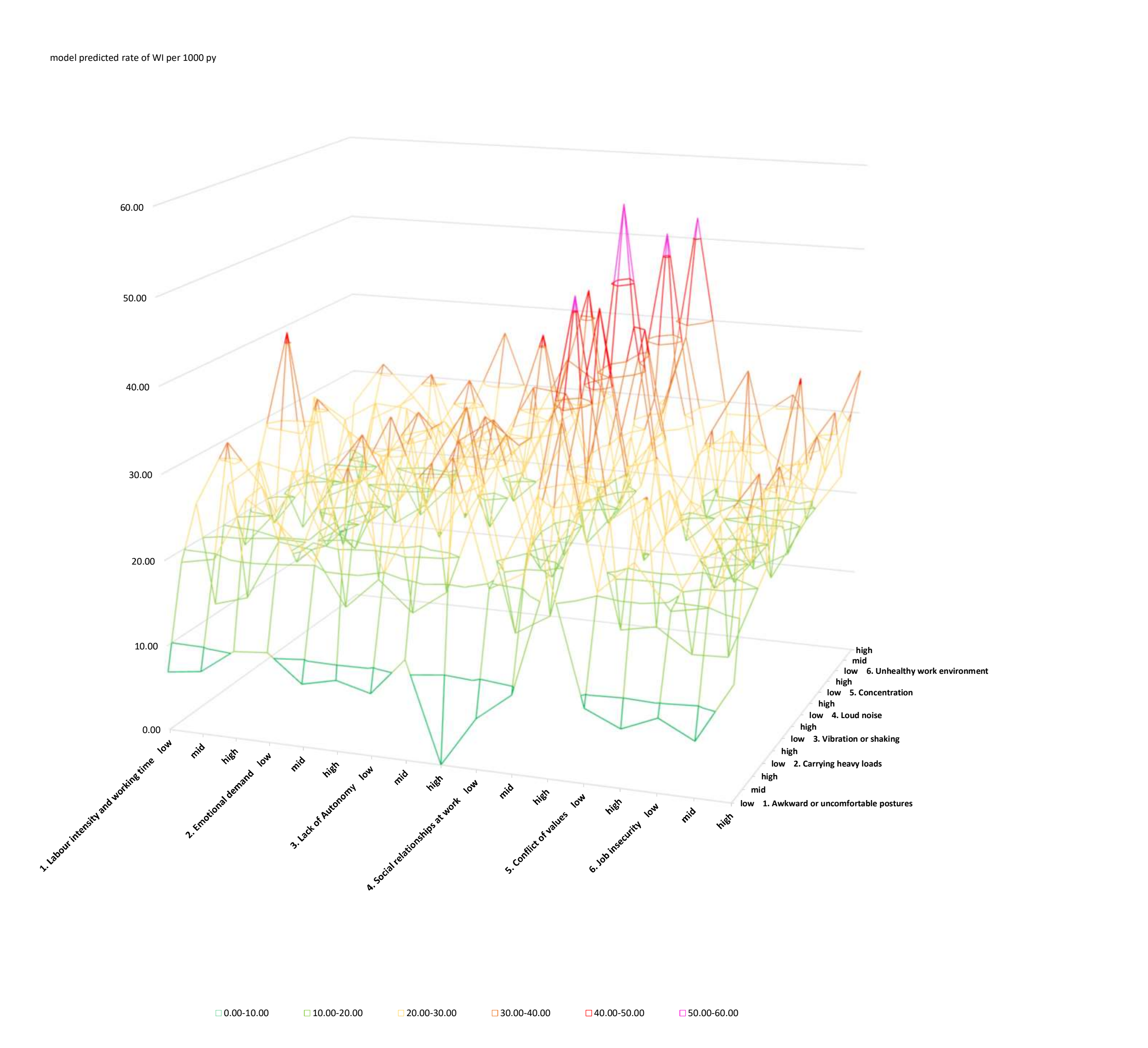

Supplement: Supplementary file 5 [file Data_Sheet_5.PDF]
